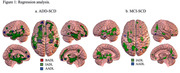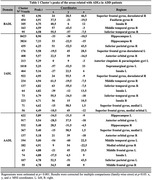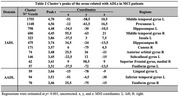# Neuroanatomical Correlates of Functional Capacity in Activities of Daily Living in the Alzheimer´s Disease Continuum

**DOI:** 10.1002/alz.089192

**Published:** 2025-01-09

**Authors:** Fernando Henriquez, Patricio Riquelme Contreras, Cecilia Gonzalez Campo, Patricia Lillo, Daniela Thumala, David Martínez‐Pernía, Cecilia Okuma, Michele Demanet, Rodrigo Saguez, Joaquin Herrero, Rodrigo Henriquez, Francisco Aboitiz, Andrea Slachevsky Chonchol

**Affiliations:** ^1^ Neuropsychology and Clinical Neuroscience Laboratory (LANNEC), Physiopathology Department ‐ ICBM, Neuroscience and East Neuroscience Departments, Faculty of Medicine, Universidad de Chile, Santiago Chile; ^2^ Interdisciplinary Center for Neuroscience (NeuroUC) ‐ Laboratory for Cognitive and Evolutionary Neuroscience ‐ Medicine School ‐ Pontificia Universidad Católica de Chile, Santiago Chile; ^3^ Geroscience Center for Brain Health and Metabolism (GERO), Santiago Chile; ^4^ Department of Medical Technology. Faculty of Medicine. Universidad de Chile, Santiago Chile; ^5^ Memory and Neuropsychiatric Center (CMYN), Neurology Department, Hospital del Salvador and Faculty of Medicine, Universidad de Chile, Santiago Chile; ^6^ Masters in biological sciences / Neurosciences. Universidad de Valparaíso, Chile., Valparaíso Chile; ^7^ CONICET, Buenos Aires Argentina; ^8^ Cognitive Neuroscience Center (CNC), Universidad de San Andrés, Buenos Aires, Buenos Aires Argentina; ^9^ Neurology Unit, Hospital San José, Santiago Chile; ^10^ East Neuroscience Departments, Faculty of Medicine, University of Chile, Santiago Chile; ^11^ Interuniversity Center on Healthy Aging, Santiago Chile; ^12^ Department of Psychology, University of Chile, Santiago Chile; ^13^ Center for Social and Cognitive Neuroscience (CSCN), School of Psychology, Universidad Adolfo Ibáñez, Santiago Chile; ^14^ Instituto de Neurocirugía Dr. Alfonso Asenjo, Santiago Chile; ^15^ School of Kinesiology, Faculty of Medicine, Universidad Finis Terrae, Santiago Chile; ^16^ Neurology Service, Department of Medicine, Clínica Alemana‐Universidad del Desarrollo, Santiago, Chile., Santiago Chile

## Abstract

**Background:**

The Alzheimer's Disease (AD) continuum is composed of Subjective Cognitive Decline (SCD), Mild Cognitive Impairment (MCI), and Alzheimer's Disease Dementia (ADD). Changes in grey matter volume (GMV), characteristic of the AD continuum, are related to cognitive and activities of daily living (ADL) impairments. ADLs are divided into three domains: i) Basic (BADL), ii) Instrumental (IADL), and iii) Advanced (AADL), and their study is critical for understanding the evolution and adequate follow‐up of patients. To date, the neuroanatomical basis of impairment in ADL has not been addressed. This work aimed to study the relationship between GMV and the ADL domains in the AD continuum.

**Method:**

A cross‐sectional study of 77 SCD, 30 MCI, and 23 ADD, matched for age, sex, and education, was conducted. ADLs were assessed with the Technology‐Activities of Daily Living Questionnaire (T‐ADLQ). A voxel‐wise regression analysis (with the SPM module) was performed to explore the association between GMV and the domains of ADLs. Total Intracranial Volume (TIV) was entered as a covariate. The analysis was performed for each patient group (MCI and ADD) in tandem with the SCD group to increase sample size, data variance, and statistical power.

**Result:**

Regression analysis for ADD‐SCD (Figure 1a) shows that in ADD patients (Table 1), AADLs were associated with decreased GMV in temporal, frontal, parietal, and insular regions. IADLs were also associated with temporal, frontal, parietal, and insular areas but were more widely distributed. BADLs were associated with temporal, frontal, and insular structures. The MCI‐SCD analysis (Figure 1b) shows that in patients with MCI (Table 2), AADLs were associated with temporal areas and IADLs with temporal, frontal, and parietal regions. BADLs had no significant associations.

**Conclusion:**

Associations between ADL domains with decreased GMV were more widely distributed in ADD compared to the MCI group, with a pattern similar to the neurodegenerative progression in the AD continuum. This finding highlights the potential of diverse neuroanatomical markers of functional capacity at different stages of the AD continuum. Such markers could hold significant relevance for the classification and follow‐up of patients from an ADL perspective, thereby improving the understanding of the consequences in daily life.